# Three-dimensional finite element analysis of tooth and implant-supported telescopic prosthesis using zirconia, PEKK, and cobalt chromium crowns

**DOI:** 10.1186/s12903-025-07607-6

**Published:** 2026-01-26

**Authors:** Sarah Zaman Sahib Awad, Khloud Ezzat Mourad, Ahmed Sameh, Ahmed Heji Albaqawi, Aisha Zakaria Hashem Mostafa

**Affiliations:** 1 Prosthodontic Department, Faculty of Dentistry, Mansoura University, Mansoura City, Egypt; 2 Department of Removable Prosthodontics, Faculty of Dentistry, Mansoura University, Mansoura, Egypt; 3 Department of Prosthodontics, Faculty of Dentistry, Mansoura National University, Gamasa City, Egypt; 4 Production Engineering and Mechanical Design Department, Faculty of Engineering, Mansoura University, Mansoura, Egypt; 5 Mechatronics Department, Faculty of Engineering, Horus University, New Damietta, Egypt; 6 Department of Restorative Dental Science, College of Dentistry, University of Haˈil, Haˈil, Makkah, Kingdom of Saudi Arabia; 7 Prosthodontic Department, Faculty of Dentistry, Mansoura National University, Gamasa City, Egypt

**Keywords:** Finite element analysis, Telescopic prosthesis, PEKK, Zirconia, Cobalt-chromium alloy, Prosthodontic biomechanics

## Abstract

**Objectives:**

This study aimed to evaluate the biomechanical behavior and stress distribution of tooth–implant-supported telescopic prostheses using different combinations of zirconia, polyetherketoneketone (PEKK), and cobalt–chromium (Co-Cr) materials for primary and secondary crowns by means of three-dimensional finite element analysis (3D FEA).

**Materials and methods:**

A three-dimensional finite element model representing a mandibular arch restored with a telescopic overdenture supported by two canines and two implants in the molar region was constructed. Nine prosthetic configurations were analyzed based on different primary and secondary crown material combinations (zirconia, PEKK, and Co-Cr). All materials were assumed to be homogeneous, isotropic, and linearly elastic. Static axial occlusal loads simulating centric occlusion were applied. Von Mises stress distribution was evaluated in prosthetic components, implants, natural teeth, and surrounding bone.

**Results:**

Material selection significantly influenced stress distribution within the telescopic system and supporting structures. PEKK used as a secondary crown reduced stress concentrations within the prosthetic crowns but resulted in increased stress transfer to the supporting bone, teeth, and implants compared with zirconia and Co-Cr. When PEKK was used as a primary crown, stress levels within prosthetic components were reduced, while stress transmission to supporting structures was not substantially altered. Zirconia and Co-Cr demonstrated comparable biomechanical behavior across all configurations. In all models, secondary crowns exhibited higher stress values than primary crowns, and implants were subjected to greater loads than natural teeth.

**Conclusions:**

Within the limitations of this 3D finite element study, material combinations in tooth–implant-supported telescopic prostheses significantly affect biomechanical stress distribution. PEKK exhibits a material-dependent stress-modulating behavior that should be carefully considered during prosthetic design to balance prosthetic protection and load transfer to supporting structures.

## Introduction

Prosthetic rehabilitation of partially edentulous patients with a limited number of remaining teeth presents a clinical challenge that requires careful biomechanical planning. Such cases may be effectively managed through the strategic placement of dental implants combined with removable prosthetic solutions, including implant-assisted removable partial dentures and overdentures retained by double-crown (telescopic) systems [1–7]. Telescopic attachments facilitate vertical load transfer and improve prosthesis stability by connecting remaining natural teeth and/or implants, thereby contributing to favorable stress distribution and long-term prosthetic performance [8, 9].

Telescopic attachment systems are widely used in tooth- and implant-supported overdentures due to their versatility and favorable clinical characteristics. These systems offer improved esthetics with reduced implant numbers, flexibility in implant positioning, ease of oral hygiene maintenance, enhanced retention and stability, and improved load distribution with reduced torque on abutments [10, 11]. A telescopic system consists of a primary crown fixed to the abutment and a secondary crown incorporated into the removable prosthesis, with retention primarily achieved through frictional contact between the two components.

Advances in digital dentistry have significantly influenced the fabrication of telescopic prostheses. Computer-aided design and computer-aided manufacturing (CAD/CAM) technologies enable precise and reproducible fabrication of double crowns through subtractive milling and additive manufacturing techniques [12, 13]. These technologies have expanded the range of available materials, allowing the clinical use of advanced ceramics and high-performance polymers with improved mechanical and biological properties [14–16].

Cobalt–chromium (Co-Cr) alloys have traditionally been used for telescopic systems because of their high strength and rigidity; however, their use is associated with esthetic limitations, high thermal conductivity, potential hypersensitivity reactions, and susceptibility to galvanic corrosion [7–18]. Ceramic materials, particularly zirconia, have gained popularity due to their superior esthetics, favorable biocompatibility, and excellent mechanical properties, making them suitable for use in primary crowns, implant abutments, and monolithic restorations [19, 20]. More recently, polyetherketoneketone (PEKK), a high-performance polymer, has emerged as a promising alternative for telescopic prostheses due to its favorable mechanical behavior, hydrolysis resistance, and biocompatibility [9, 21, 22]. PEKK exhibits a semi-crystalline structure and can be processed using digital workflows without altering its chemical properties, enabling its application in CAD/CAM-fabricated prosthetic components.

Finite element analysis (FEA) has been extensively applied in prosthodontics to investigate stress distribution and deformation in dental implants, removable and fixed prostheses, and combined tooth–implant-supported systems [23–29]. By enabling detailed simulation of complex anatomical and prosthetic structures under controlled loading conditions, three-dimensional FEA allows assessment of internal stress patterns that cannot be directly measured in vivo or adequately captured using traditional experimental methods.

Despite the increasing clinical use of telescopic attachments connecting natural teeth and implants, limited data are available regarding the biomechanical influence of different material combinations used for primary and secondary crowns. In particular, the effect of combining zirconia, PEKK, and Co-Cr materials on stress transmission within telescopic systems and supporting structures remains insufficiently investigated [30–36]. Therefore, this study aimed to evaluate the stress distribution and biomechanical behavior of tooth–implant-supported telescopic prostheses using various combinations of zirconia, PEKK, and cobalt–chromium crowns through three-dimensional finite element analysis. The null hypothesis was that the stresses transmitted to prosthetic components and supporting structures would not differ among the evaluated material combinations.

## Materials and methods

### Study design

This study employed a three-dimensional finite element analysis (3D FEA) approach to evaluate stress distribution in tooth–implant-supported telescopic prostheses using different material combinations for primary and secondary crowns. A mandibular model representing a partially edentulous condition with two canines and two implants in the molar region was selected to reflect a clinically relevant prosthetic scenario. Nine distinct prosthetic configurations were created based on combinations of zirconia, polyetherketoneketone (PEKK), and cobalt–chromium (Co-Cr) alloys used for the primary and secondary crowns (Table 1).

**Table 1.**
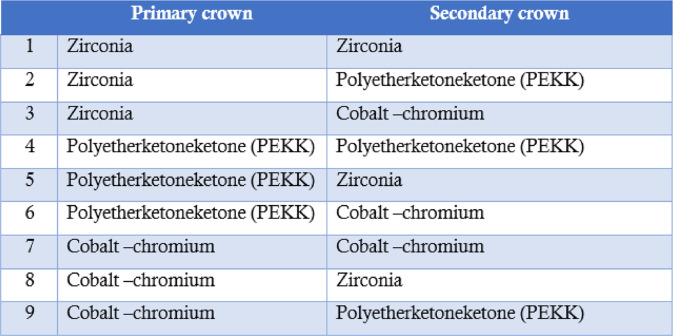
The nine different groups based on the primary and secondary crown

### Three-Dimensional model construction

Three-dimensional geometric models of the mandibular bone, natural teeth, dental implants, and telescopic crown assemblies were generated using SolidWorks 2023 Premium (Dassault Systèmes, France). The anatomical structures were constructed based on segmented computed tomography data and established anatomical landmarks to ensure realistic geometry.

**Fig. 1:**
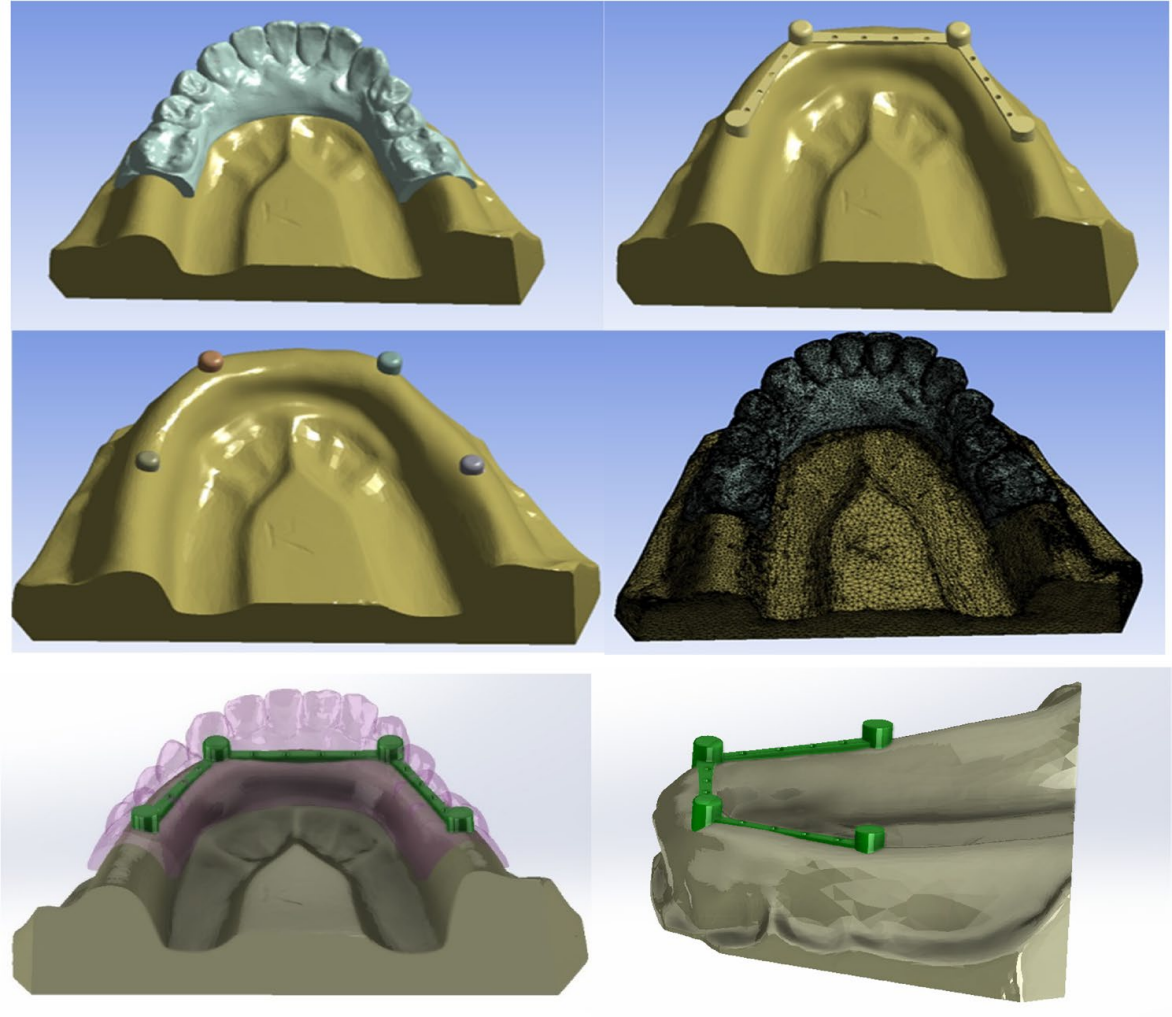
The 3D models of the prosthetic components including teeth, implants, and double-crown systems

The telescopic system consisted of a primary crown fixed to the abutment and a secondary crown integrated into the removable prosthesis. The primary crown was designed with a total taper angle of 4° (2° per wall), an occlusal thickness of 1.0 mm, and an axial wall thickness of 0.5 mm. The secondary crown was designed with an occlusal thickness of 0.8 mm and a cement space of 0.1 mm. The vertical height of the crown assembly was standardized at 6 mm (Fig. 1). These parameters were selected based on previously validated telescopic prosthesis designs [33, 34].

### Finite element meshing

The constructed models were imported into ANSYS Workbench 2019 R3 (ANSYS Inc., Canonsburg, PA, USA) for finite element discretization. A three-dimensional tetrahedral mesh using SOLID187 elements was generated. Mesh controls were standardized across all models to ensure consistency, with smoothing set to medium, a transition ratio of 0.272, a maximum of five layers, and a growth rate of 1.2.

The final finite element model comprised 736,006 nodes and 462,183 elements. Mesh convergence was verified by refining the element size from 1.0 mm to 0.8 mm and 0.6 mm, resulting in less than 3% variation in maximum von Mises stress values, confirming numerical stability.

### Material properties

Material properties assigned to bone, tooth structure, titanium implants, zirconia, PEKK, and cobalt–chromium were obtained from validated experimental studies and widely cited literature sources (Table 2). All materials were assumed to be homogeneous, isotropic, and linearly elastic to facilitate numerical stability and allow direct comparison among material combinations, consistent with standard practices in prosthodontic finite element studies.

**Table 2.**
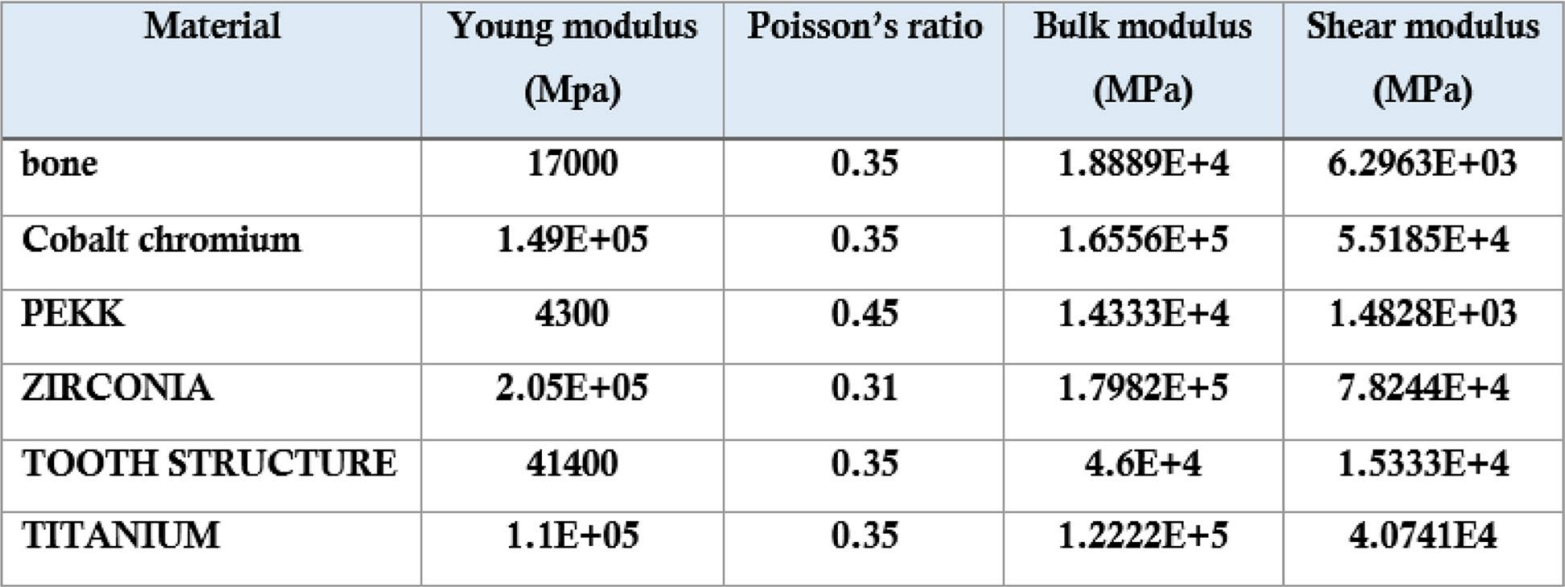
Material properties used in the study.

Table 2 shows Elastic modulus, Poisson’s ratio, shear modulus, and bulk modulus for bone, tooth structure, titanium, zirconia, PEKK, and cobalt-chromium obtained from validated literature sources

### Boundary conditions and loading protocol

The inferior border of the mandibular model was fully constrained in all translational and rotational degrees of freedom to prevent rigid-body motion and simulate fixation at temporomandibular and muscular attachment regions. Static axial occlusal loads were applied to represent centric occlusion conditions: a vertical load of 100 N was applied to each canine, and bilateral vertical loads of 250 N were applied to the molar regions.

The magnitude and direction of applied loads were selected based on reported average physiological bite forces and were applied perpendicular to the occlusal plane. All contacts between primary and secondary crowns were defined as frictional to enable realistic load transfer while preventing interpenetration.

### Finite element simulation and outcome measures

**Fig. 2:**
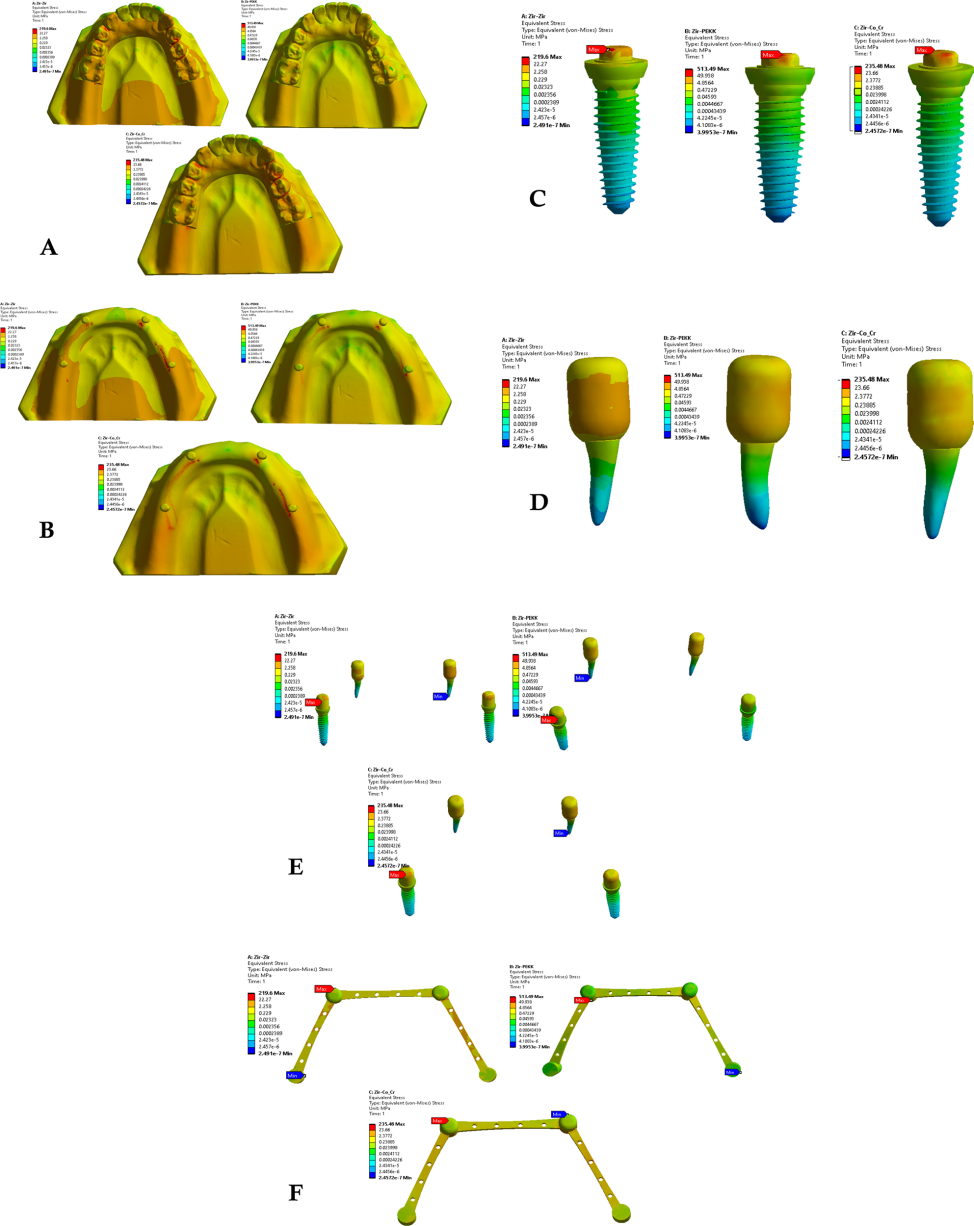
Von Mises stresses for the first 3 groups of the primary coping zirconia and secondary copy

**Fig. 3:**
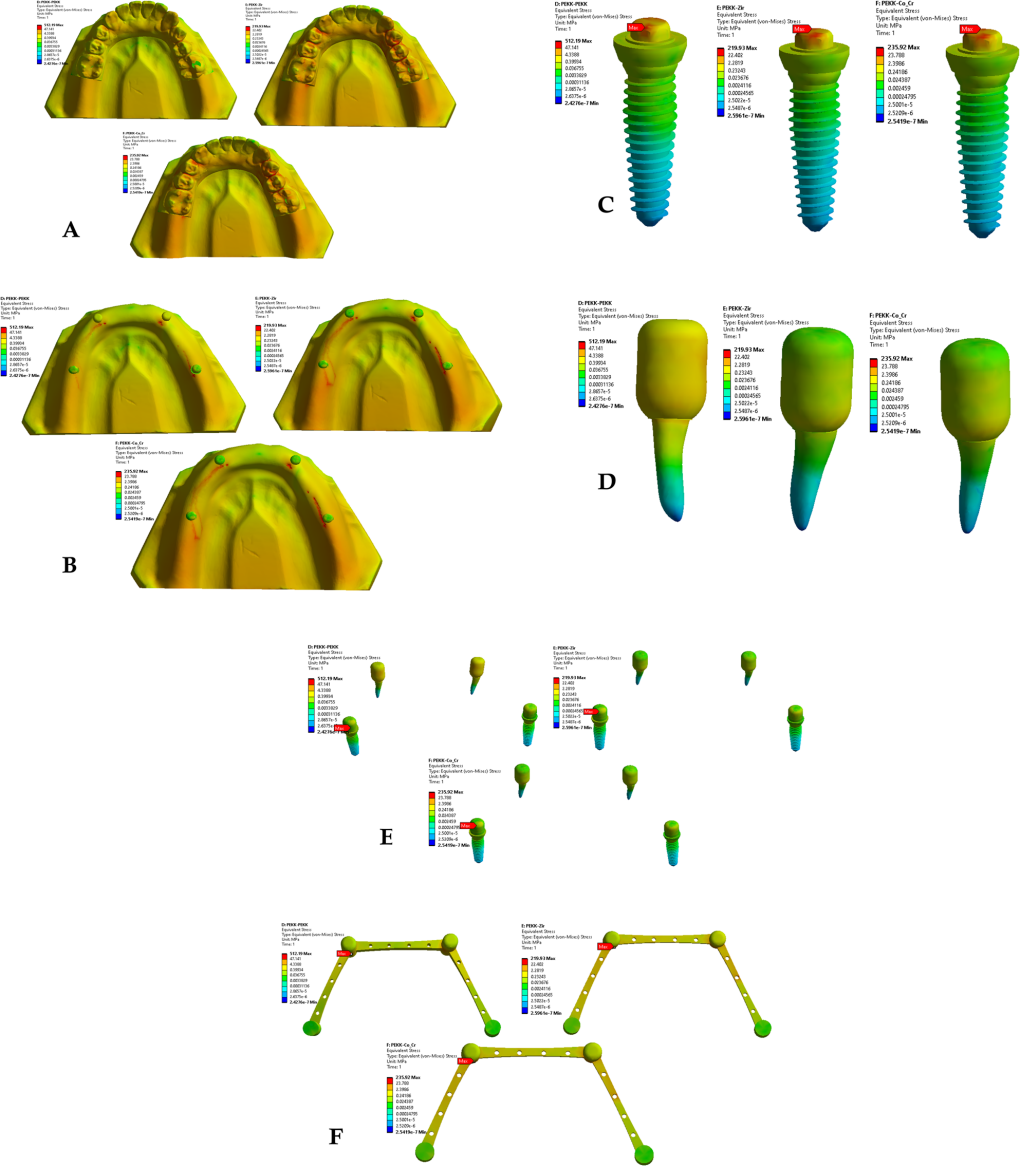
Von mises stresses for the first 3 groups of the primary coping PEKK and secondary copy zirconia, PEKK, cobalt

**Fig. 4:**
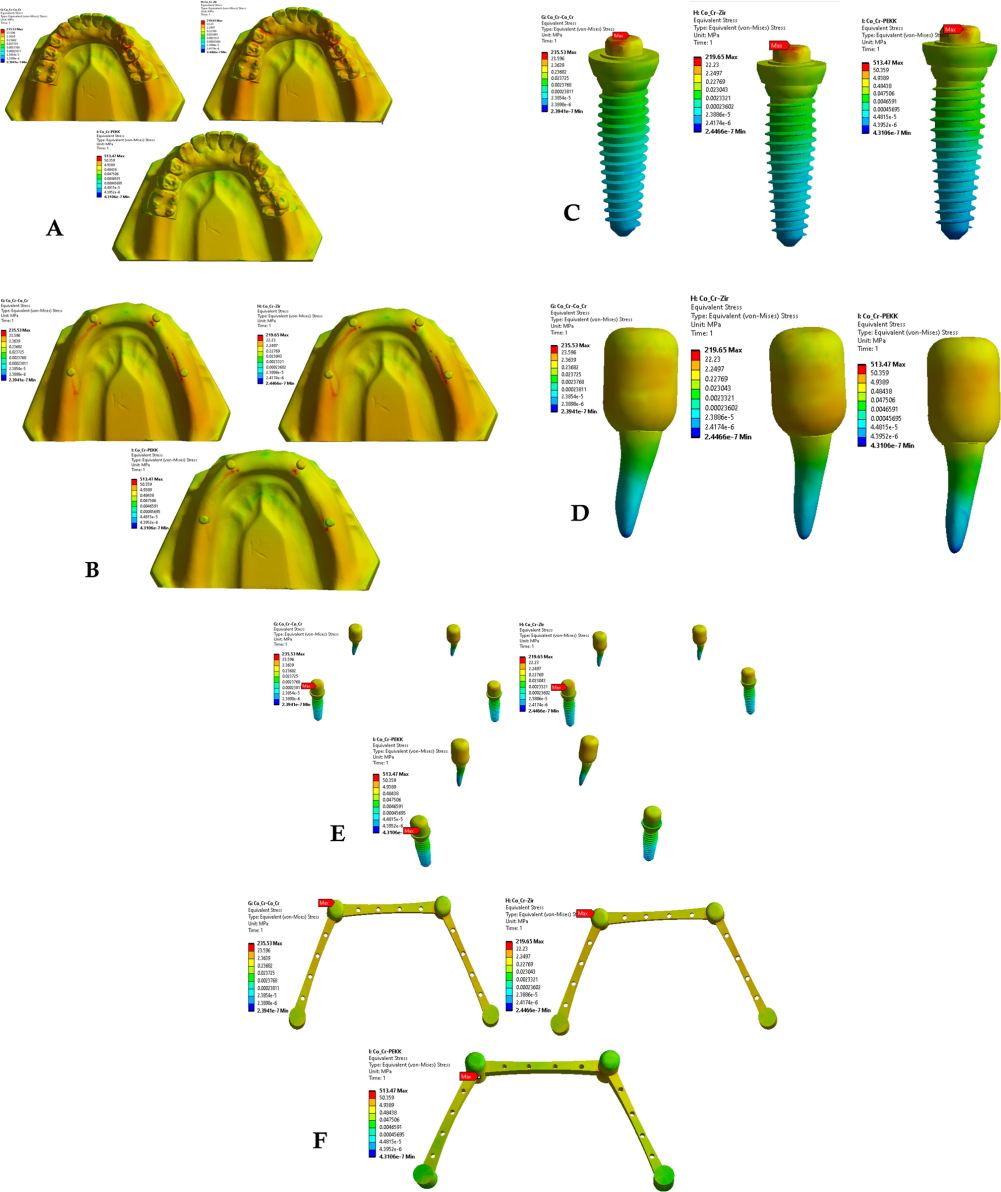
Von mises stresses for the first 3 groups of the primary coping cobalt chromium and secondary copy

Finite element simulations were conducted using ANSYS Workbench 2019 R3 for each of the nine prosthetic configurations. Von Mises stress distribution was evaluated within the primary and secondary crowns, dental implants, natural teeth, and surrounding alveolar bone. Stress patterns and peak stress values were recorded and compared across material combinations to assess the biomechanical influence of crown material selection (Figs. 2, 3 and 4).

This study was carried out at the Department of Prosthodontics, Faculty of Dentistry, Mansoura University. Ethical approval was obtained prior to commencement (Approval Code: A06012024 RP).

## Result

### Stress distribution in primary and secondary crowns

Across all material combinations, stress concentrations were predominantly observed at the occlusal surfaces and cervical regions of both primary and secondary crowns. Secondary crowns consistently exhibited higher von Mises stress values than primary crowns under identical loading conditions.

When PEKK was used as a secondary crown, peak stresses within both primary and secondary crowns were reduced compared with zirconia and cobalt–chromium secondary crowns, regardless of the primary crown material. In contrast, zirconia and cobalt–chromium secondary crowns demonstrated comparable stress magnitudes and distribution patterns.

When PEKK was used as a primary crown, stress within the prosthetic components was lower than that observed for zirconia or cobalt–chromium primary crowns; however, this configuration did not substantially alter stress transmission to the supporting structures.

### Stress distribution in supporting structures

Stress within the dental implants was consistently higher than that observed in the natural abutment teeth across all prosthetic configurations. Peak stress concentrations were primarily localized at the implant neck region and the crestal bone interface.

Use of PEKK as a secondary crown resulted in increased stress transfer to the supporting structures, including alveolar bone, implants, and natural teeth, compared with zirconia and cobalt–chromium secondary crowns. In contrast, zirconia and cobalt–chromium demonstrated.

similar biomechanical behavior with respect to stress transmission to the supporting tissues.

### Effect of material combination on load transfer

Comparative analysis of all nine material combinations demonstrated that crown material selection significantly influenced load distribution within the prosthetic system. While PEKK exhibited favorable stress-reducing behavior within prosthetic components, its lower elastic modulus promoted greater stress transfer to biological structures when used as a secondary crown.

Zirconia and cobalt–chromium, whether used as primary or secondary crowns, produced comparable stress distributions within both prosthetic and supporting components, indicating similar biomechanical performance under static axial loading conditions.

### Summary of key findings

A synthesized comparison of peak von Mises stress values across prosthetic and supporting components is presented in Tables 3, 4 and 5. Representative stress contour plots illustrating typical stress patterns for the evaluated material combinations are shown in Figs. 2, 3, 4, 5, 6, 7, 8, 9 and 10.

**Fig. 5:**
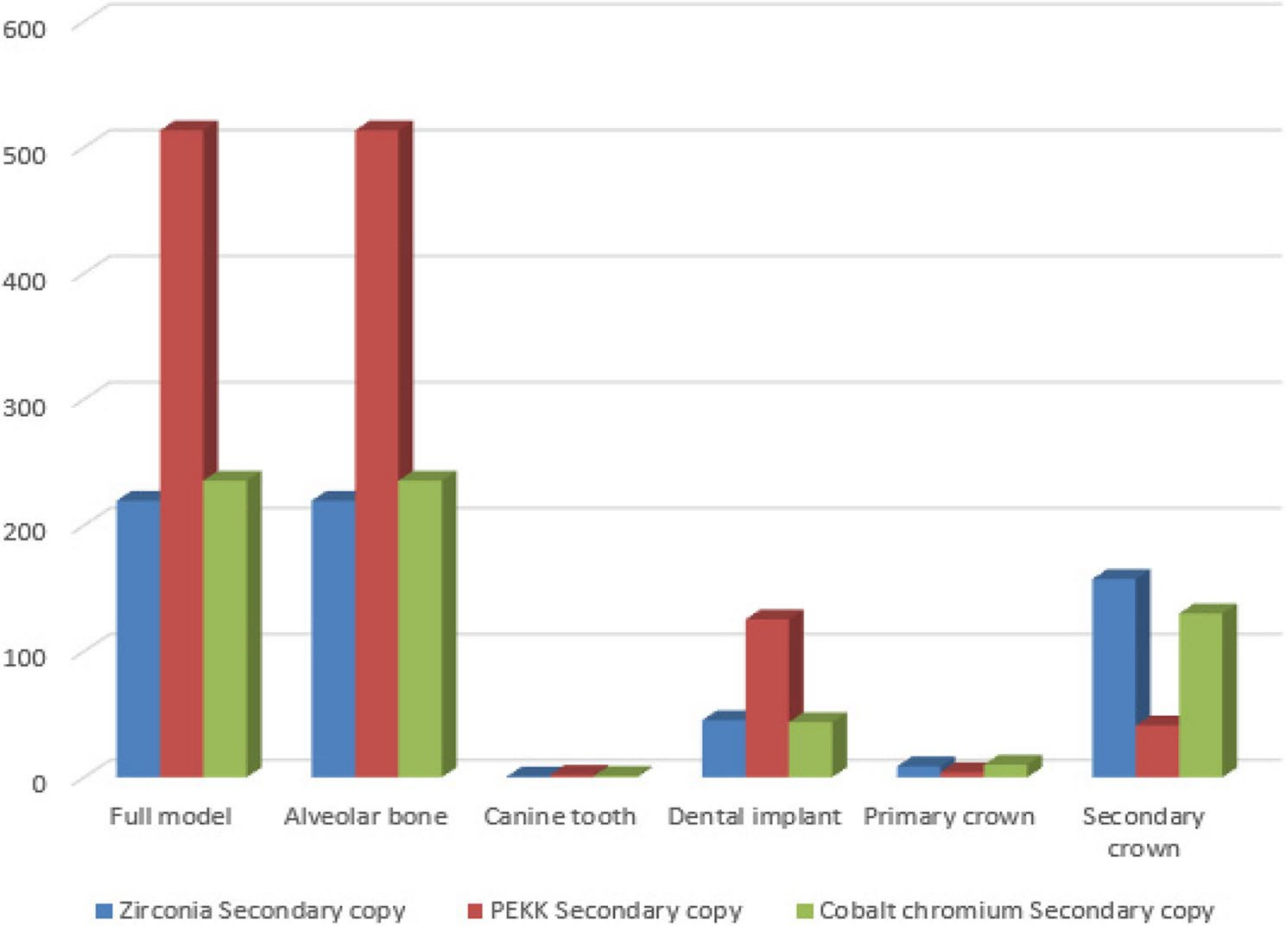
The bar chart of the maximum values of von Mises stress (Mpa) for the first 3 groups of the primary coping zirconia using 3D Finite element analysis

**Fig. 6:**
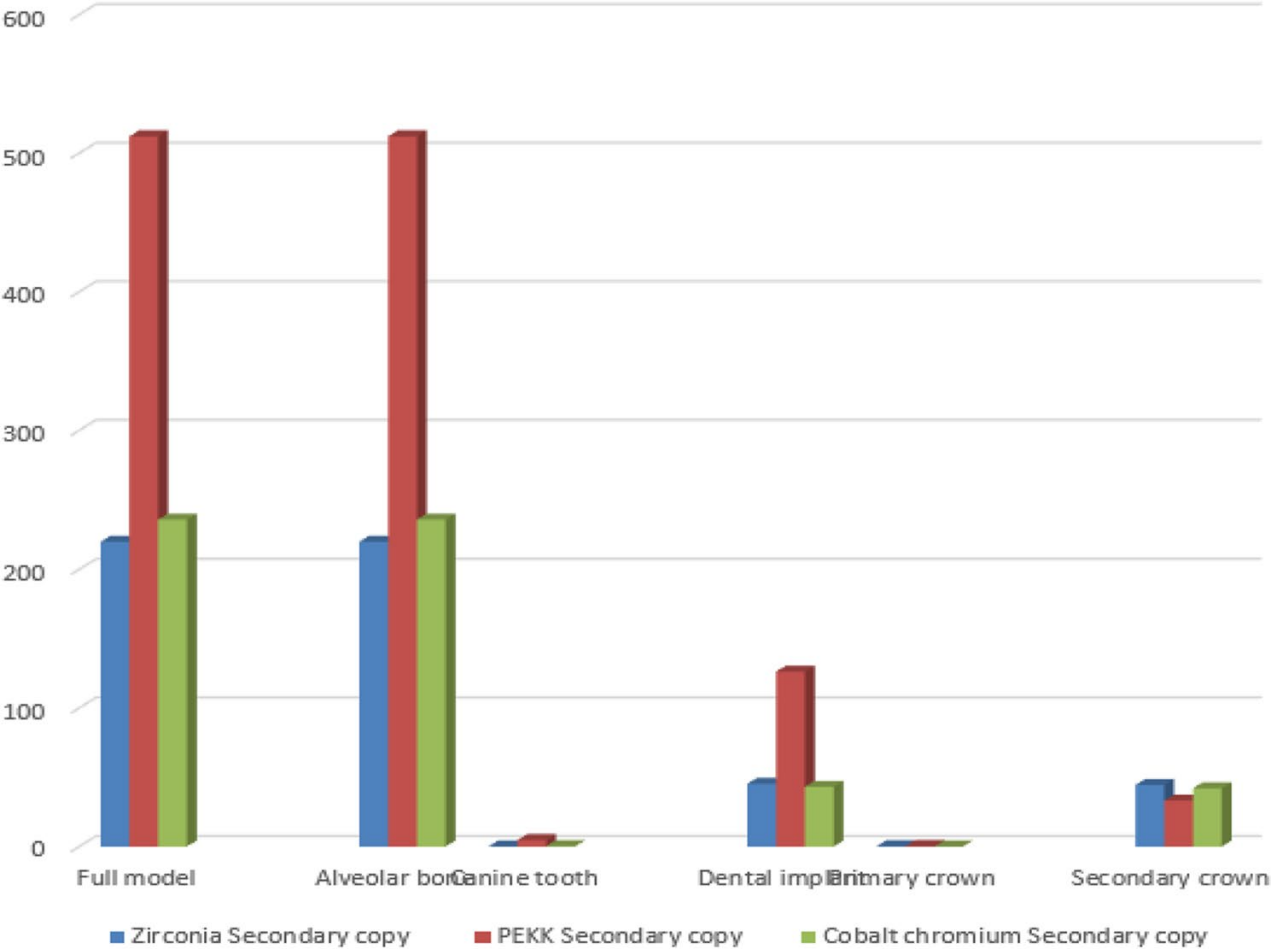
The bar chart of the maximum values of von Mises stress (Mpa) for the second 3 groups of the primary coping PEKK using 3D Finite element analysis

**Fig. 7:**
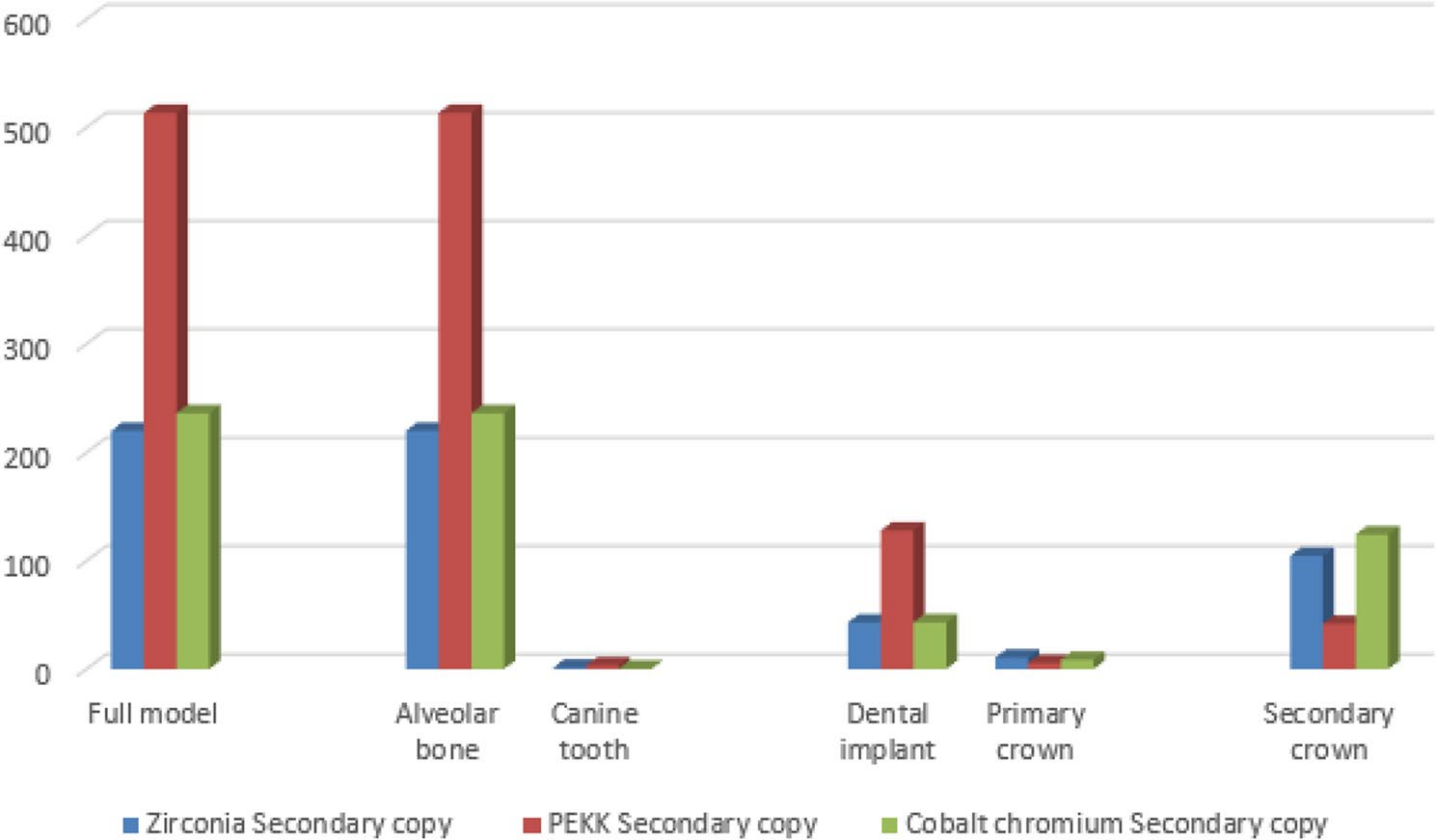
The bar chart of the maximum values of von Mises stress (Mpa) for the third 3 groups of the primary coping cobalt chromium using 3D Finite element analysis

**Fig. 8:**
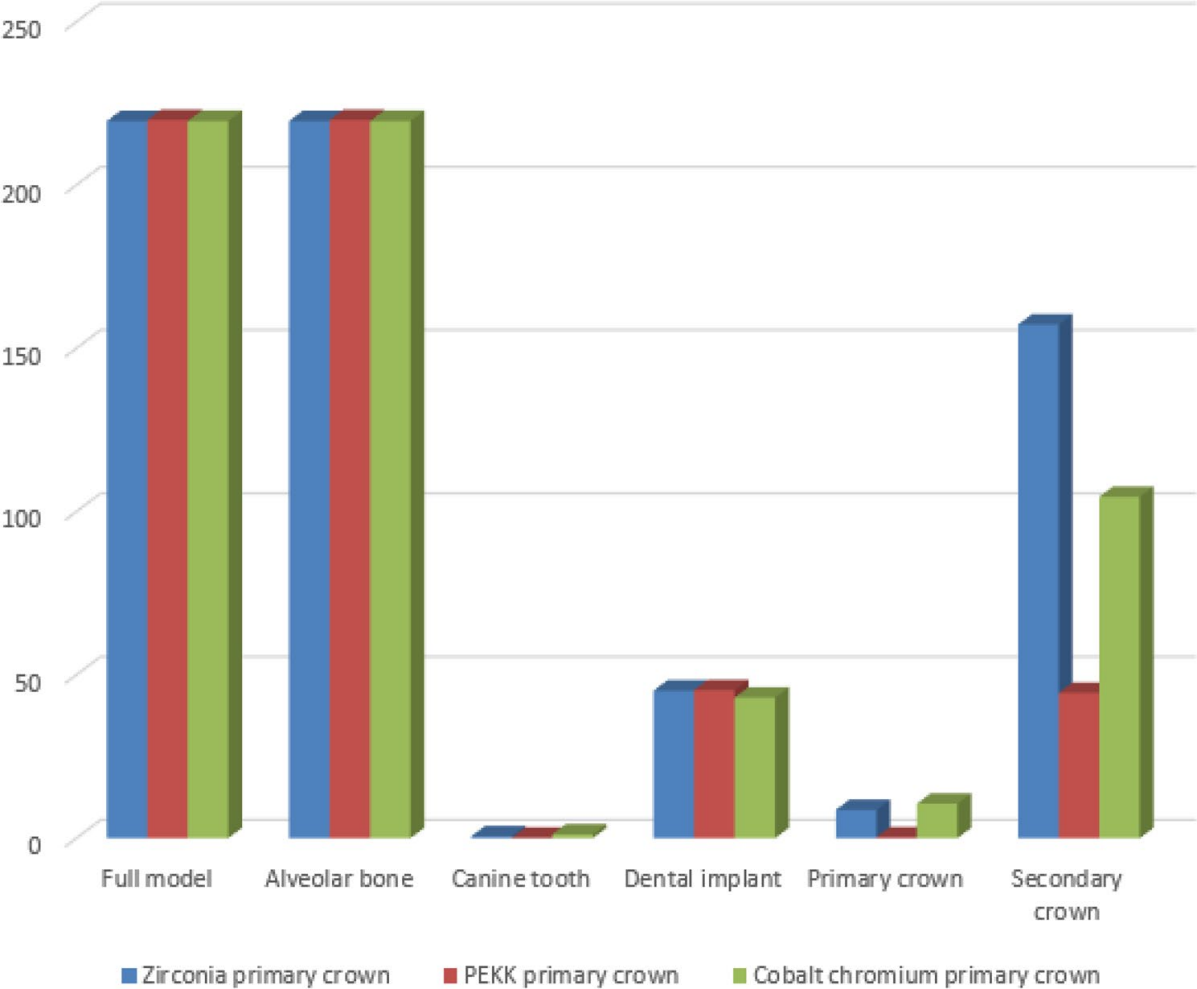
The bar chart of the maximum values of von Mises stress (Mpa) for the 3 groups of the secondary crown zirconia using 3D Finite element analysis

**Fig. 9:**
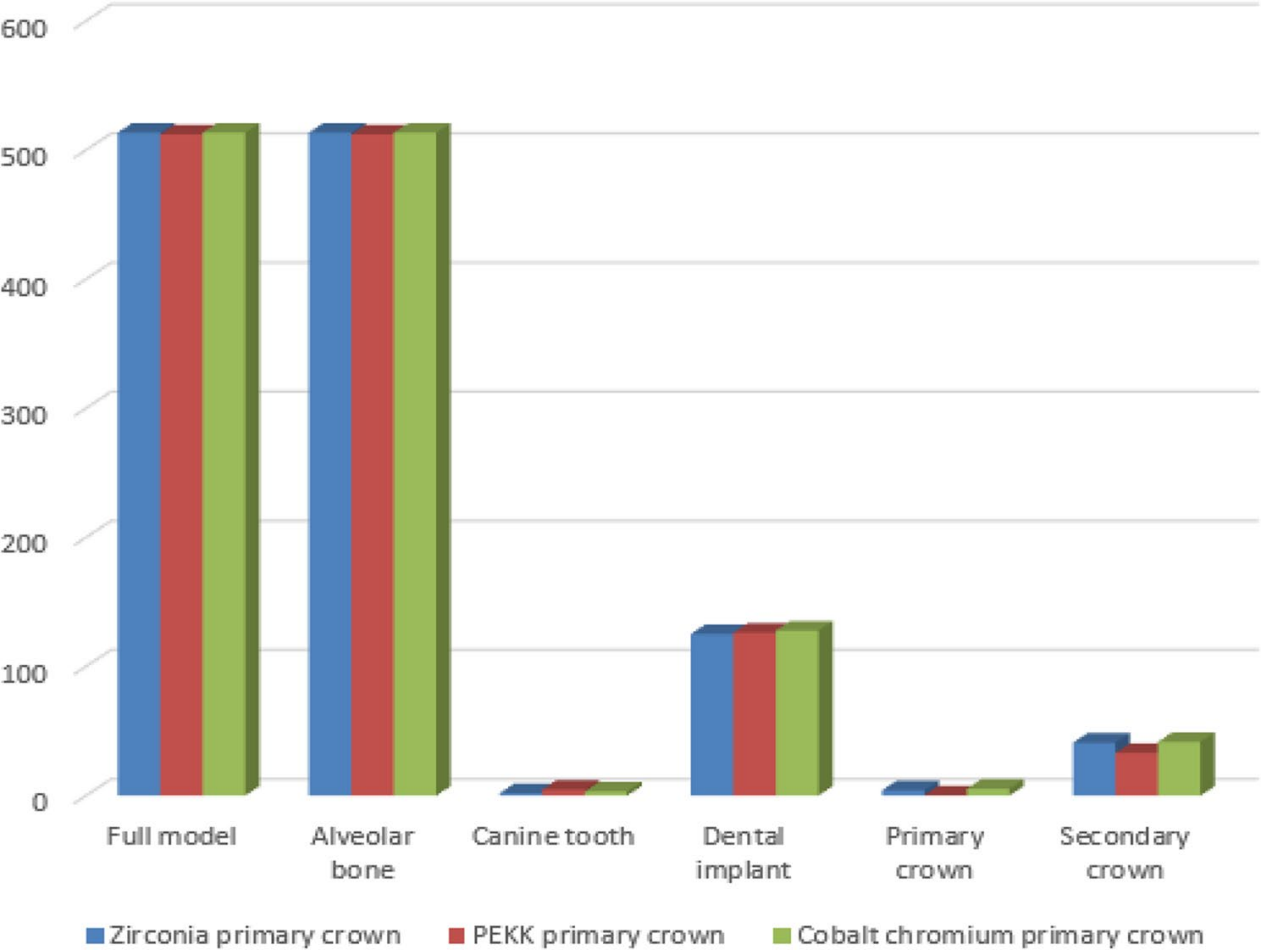
The bar chart of the maximum values of von Mises stress (Mpa) for the 3 groups of the secondary crown PEKK using 3D Finite element analysis

**Fig. 10:**
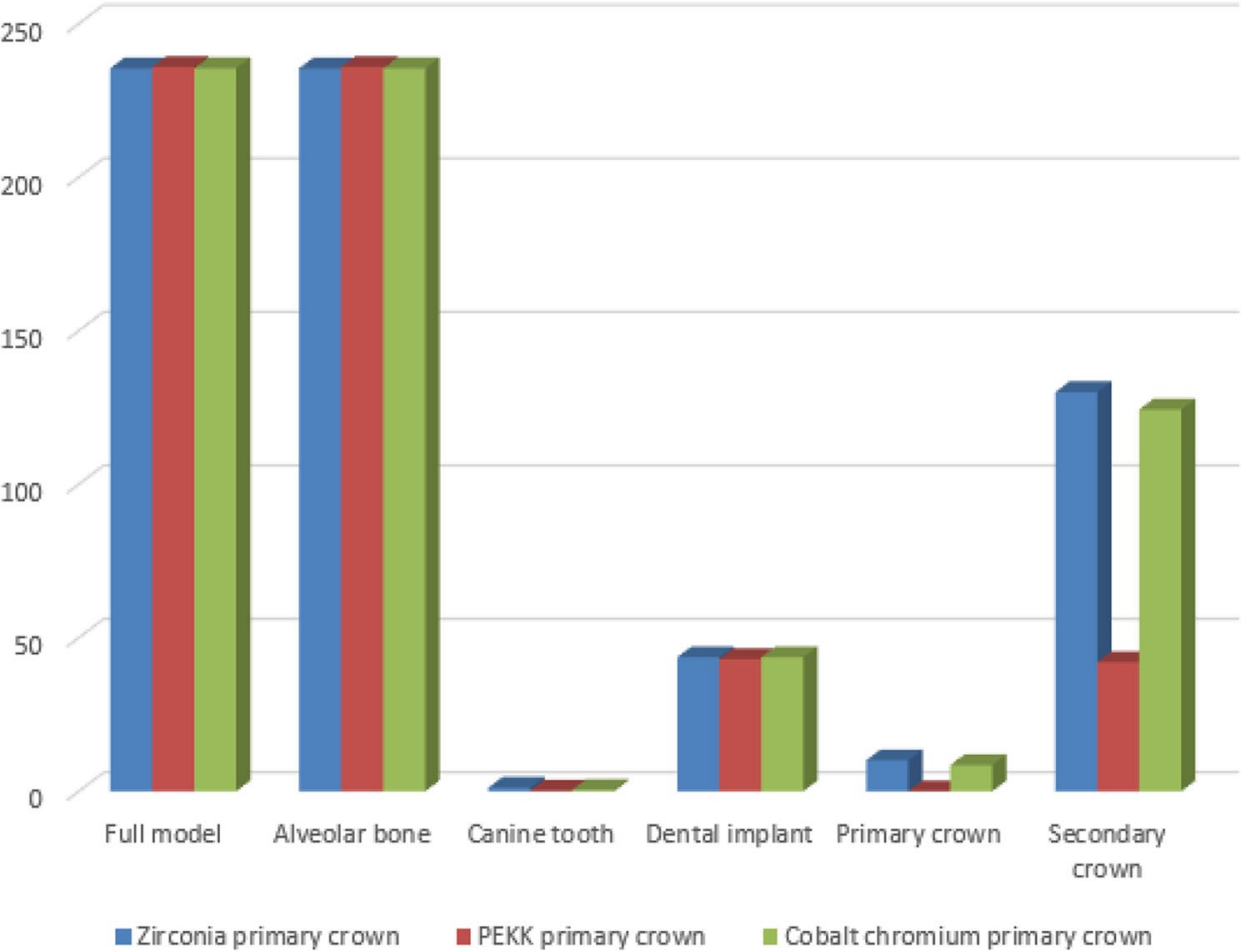
The bar chart of the maximum values of von Mises stress (Mpa) for the 3 groups of the secondary crown cobalt chromium using 3D Finite element analysis

**Table 3.**
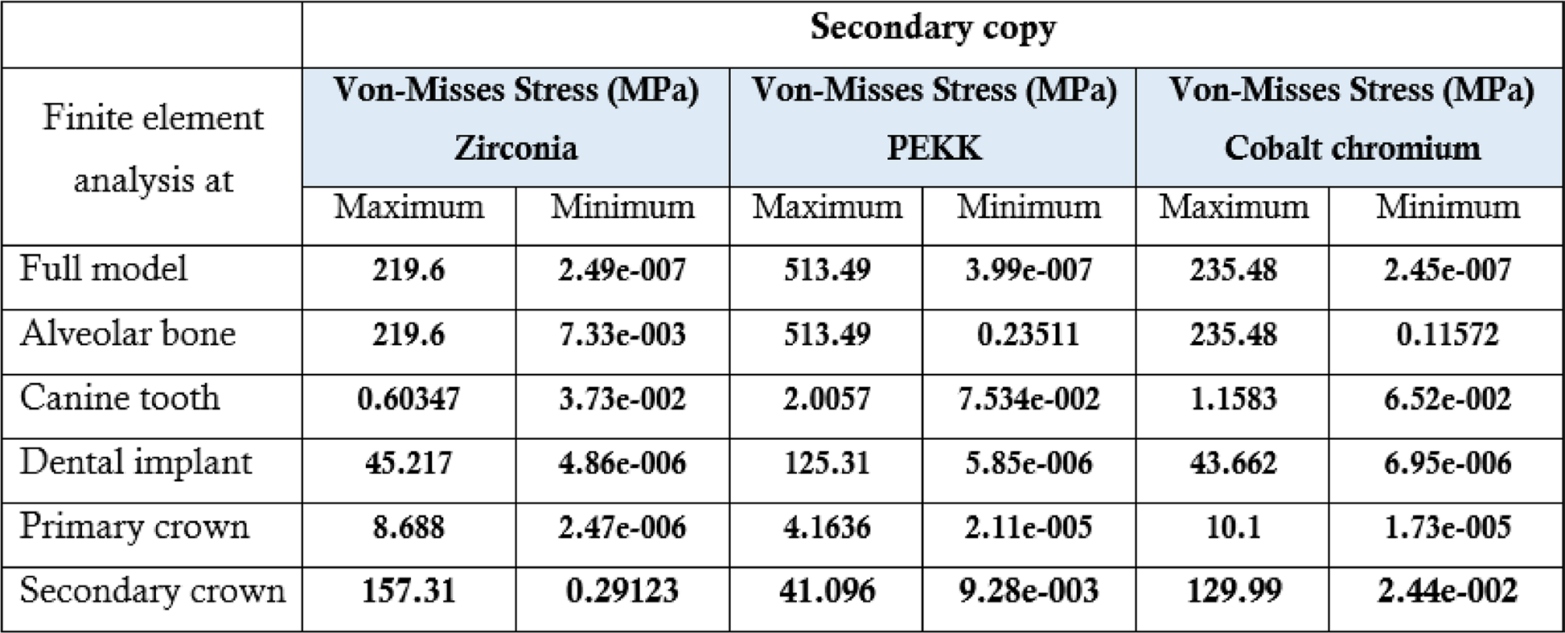
The maximum, minimum values of von Mises stress (MPa) for the first 3 groups of the primary coping zirconia using 3 D Finite element analysis at different evaluation sites

Table3 Peak stress generated in each primary crown material under standardized loading conditions for the nine telescopic prosthesis configurations

**Table 4.**
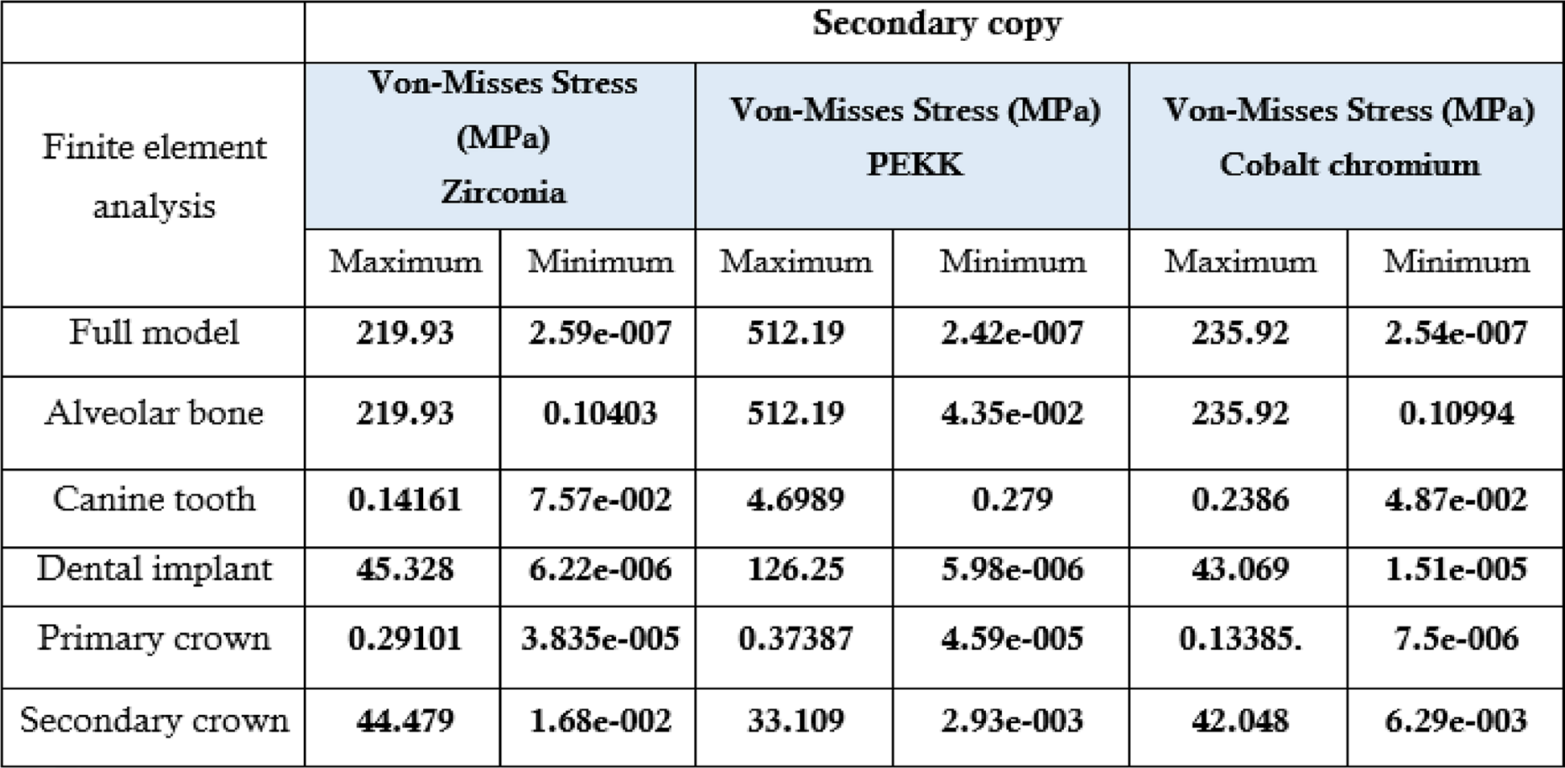
The maximum, minimum values of von mises stress (MPa) for the second 3 groups of the primary coping PEKK

Table 4 Stress distribution results showing maximum stress concentration in secondary crowns across all zirconia, PEKK, and cobalt-chromium combinations

**Table 5.**
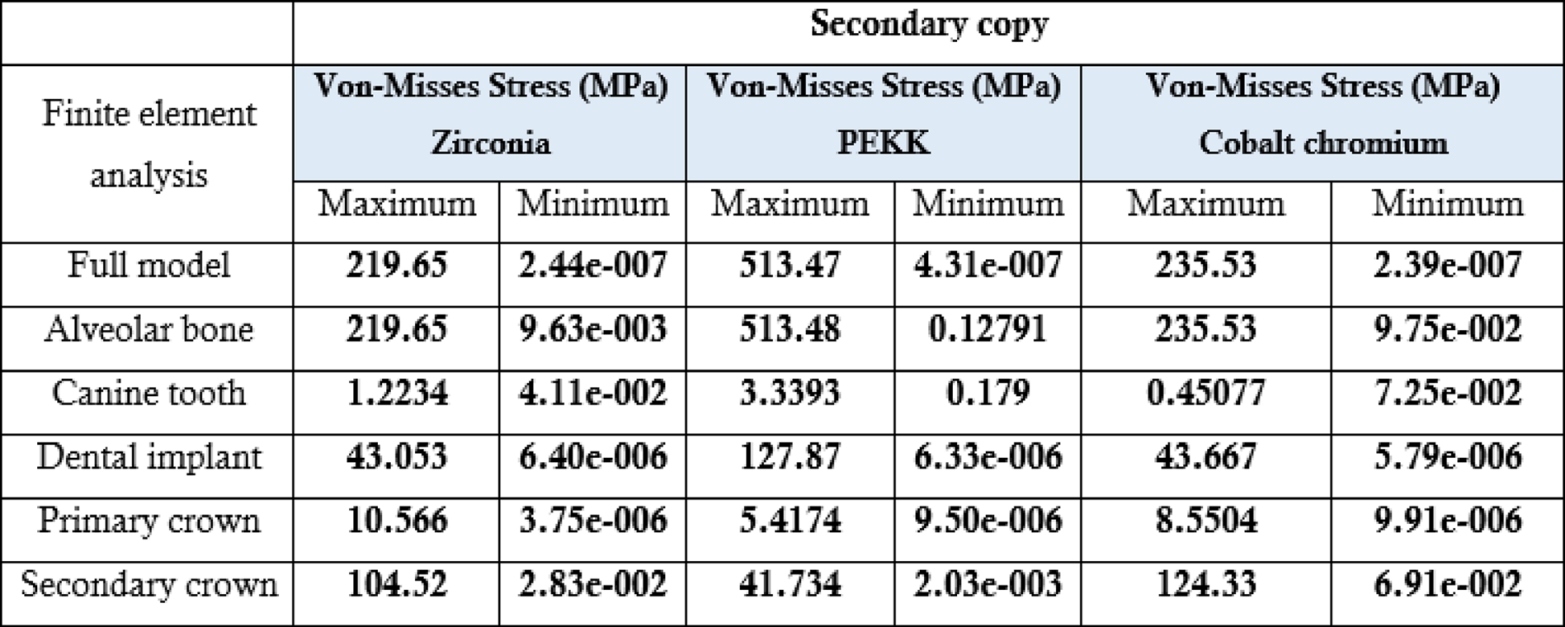
The maximum, minimum values of von Mises stress (MPa) for the third 3 groups of the primary coping cobalt chromium

Table5 Comparative analysis of stress transferred to alveolar bone, natural teeth, and implants for all tested double-crown material configurations

## Discussion

This three-dimensional finite element analysis investigated the biomechanical behavior of tooth–implant–supported telescopic prostheses fabricated using different combinations of zirconia, PEKK, and cobalt–chromium for the primary and secondary crowns. The findings demonstrate that material selection within telescopic systems significantly influences stress transfer pathways between prosthetic components and supporting biological structures. Accordingly, the null hypothesis was rejected.

Direct in vivo measurement of stress in abutment teeth, implants, and alveolar bone remains impractical due to ethical and technical constraints. Experimental techniques such as strain gauges and Photoelastic analysis provide valuable insights but are limited by localized measurement zones, material requirements, and difficulty in reproducing complex anatomical geometries [27, 37, 38]. Finite element analysis therefore represents a validated and widely accepted approach for investigating biomechanical behavior in prosthodontic systems, enabling controlled evaluation of stress distribution patterns under standardized loading conditions. Numerous experimental–numerical correlation studies have demonstrated that FEA reliably reproduces clinically relevant stress trends when appropriate material properties and boundary conditions are applied [37, 39, 40].

Previous investigations have primarily focused on single-material telescopic systems, whereas limited data exist regarding mixed-material primary–secondary crown combinations. From a clinical perspective, this distinction is important because the primary crown is usually minimally adjusted after cementation, while the secondary crown is modified to achieve optimal frictional retention and path of insertion [41]. Consequently, material pairing rather than isolated material selection may play a decisive role in long-term biomechanical performance.

The present results indicate that PEKK, when used as a secondary crown, generated higher stress levels in the supporting structures—namely alveolar bone, abutment teeth, and implants—compared with zirconia and Co–Cr secondary crowns. This behavior can be attributed to the lower elastic modulus of PEKK, which allows greater deformation at the crown interface, increasing load transfer to the underlying structures. These findings are consistent with previous experimental and numerical studies reporting higher strain levels in PEKK-based telescopic systems compared with Co–Cr alloys [33, 34]. In contrast, the substantially higher stiffness of Co–Cr (≈ 220–230 GPa) limits deformation and thereby reduces stress transmission to supporting tissues.

Frictional behavior between telescopic components further contributes to these observations. PEKK exhibits higher frictional retention compared with zirconia and Co–Cr, which enhances prosthesis stability but simultaneously increases resistance during functional loading [35, 36, 42–45]. Increased friction has been shown to elevate stress transfer within attachment systems, particularly at the implant–bone interface [42]. Therefore, while PEKK may offer favorable retention characteristics, its use as a secondary crown should be carefully considered in cases with compromised supporting structures.

Importantly, despite increasing stress in the supporting tissues, PEKK secondary crowns demonstrated lower stress concentrations within both primary and secondary crowns themselves. This apparent contradiction is resolved by considering the viscoelastic and damping properties of PEKK. The lower elastic modulus and higher energy absorption capacity of PEKK create a cushioning effect that reduces stress accumulation within the prosthetic components, even as greater loads are transferred to the supporting structures [36, 46–48]. This dual behavior explains why PEKK cannot be uniformly classified as biomechanically superior or inferior, but rather as material-dependent on its prosthetic role.

When PEKK was used as a primary crown, stresses within the prosthetic components were reduced without a significant effect on supporting structures. This finding supports previous reports describing favorable stress modulation when PEKK is used as a framework or primary element due to its elastic recovery and toughness [48–50]. In contrast, zirconia and Co–Cr exhibited similar stress distributions regardless of their position as primary or secondary crowns, reflecting their high stiffness and limited energy dissipation capacity.

Across all material combinations, secondary crowns consistently experienced higher stress than primary crowns. This observation aligns with prior FEA and experimental studies demonstrating that secondary crowns act as the primary load-receiving component during mastication and insertion–removal cycles [20, 36, 51]. Additionally, implants were subjected to higher stress levels than natural teeth, which is consistent with the absence of a periodontal ligament and its associated shock-absorbing capacity [52–56].

Localized stress peaks at the crestal cortical bone were observed around implants in all models. Such concentrations are well documented in implant FEA studies and are generally interpreted as numerical singularities resulting from geometric transitions and idealized boundary conditions rather than predictors of immediate clinical failure [57–59]. The reproduction of these stress patterns further supports the validity of the present modeling approach.

The modeling assumptions adopted in this study—homogeneous, isotropic, and linearly elastic material behavior; omission of the periodontal ligament; and application of static axial loads—are consistent with numerous previously validated dental FEA investigations [33, 36, 52–55]. Sensitivity analysis confirmed that moderate variations in elastic modulus and friction coefficients resulted in less than 3% change in peak cortical bone stress, indicating numerical stability. Nevertheless, these assumptions limit direct clinical extrapolation and should be interpreted as representing idealized loading conditions rather than exact physiological behavior.

Future investigations should incorporate dynamic loading, nonlinear material properties, periodontal ligament simulation, and patient-specific geometries to further refine biomechanical predictions. Long-term clinical studies are also necessary to evaluate the influence of wear, fatigue, and cement layer behavior on telescopic prosthesis performance.

### Limitations

The present investigation employed a three-dimensional finite element model using established and widely accepted biomechanical assumptions to enable controlled comparison between material combinations. Materials were modeled as homogeneous, isotropic, and linearly elastic, and static axial loading was applied to represent standardized occlusal conditions commonly adopted in comparable finite element studies. Periodontal ligament simulation was not incorporated in order to isolate material-dependent effects and maintain numerical stability of the mixed tooth–implant model. The analysis focused on a single mandibular telescopic prosthesis design to ensure consistency across all simulations. Accordingly, the findings should be interpreted within the context of comparative biomechanical behavior rather than direct clinical outcome prediction. Future studies may expand upon the current model by incorporating dynamic loading conditions, patient-specific geometries, and experimental validation to further refine clinical relevance. [60–64]

## Conclusion

This three-dimensional finite element analysis demonstrated that the biomechanical performance of tooth–implant–supported telescopic prostheses is strongly influenced by the material combination used for primary and secondary crowns. The results highlight that material pairing, rather than isolated material selection, plays a decisive role in governing stress distribution within both prosthetic components and supporting biological structures.

PEKK exhibited a material-dependent biomechanical behavior. When used as a secondary crown, PEKK reduced stress concentrations within the prosthetic crowns but increased stress transfer to the supporting bone, teeth, and implants. Conversely, PEKK used as a primary crown contributed to stress modulation within the prosthetic components without significantly affecting the supporting structures. Zirconia and cobalt–chromium demonstrated comparable biomechanical performance regardless of their position within the telescopic system, reflecting their high stiffness and limited damping capacity.

Across all configurations, secondary crowns consistently experienced higher stress than primary crowns, and implants were subjected to greater stress than natural teeth, underscoring the biomechanical influence of periodontal support. These findings provide mechanistic insight into stress transfer pathways in mixed tooth–implant telescopic systems and support informed material selection tailored to individual clinical conditions.

Within the constraints of a controlled numerical model, the present study contributes evidence-based guidance for optimizing telescopic prosthesis design and offers a biomechanical framework for future experimental and clinical investigations.

## Data Availability

The data sets used in the current study are available from the corresponding author upon request.

## Abbreviations

FEA: Finite element analysis
PEKK: Polyetherketoneketone
Co-Cr: Cobalt–chromium
PDL: Periodontal ligament
RPD: Removable partial denture
CAD/CAM: Computer-aided design/computer-aided manufacturing
CT: Computed tomography

## References

[1] de Freitas RF, de Carvalho Dias K, da, Porto Carreiro F, Barbosa A, Ferreira GA. MA. Mandibular implantsupported removable partial denture with distal extension: A systematic review. J Oral Rehabil. 2012;39(10):791–98. 10.1111/j.1365-2842.2012.02326.x 22882547

[2] Putra Wigianto AY, Goto T, Iwawaki Y, Ishida Y, Watanabe M, Ichikawa T. Treatment outcomes of implant-assisted removable partial denture with distal extension based on the Kennedy classification and attachment type: a systematic review. Int J Implant Dent. 2021;7(1):111. 34773513 10.1186/s40729-021-00394-z PMC8590637

[3] Kuroshima S, Ohta Y, Uto Y, Al-Omari FA, Sasaki M, Sawase T. Implant-assisted removable partial dentures: part I. a scoping review of clinical applications. J Prosthodont Res. 2024;68(1):20–39. 37164658 10.2186/jpr.JPR_D_22_00252

[4] Szentpetery V, Lautenschlager C, Setz JM. Frictional telescopic crowns in severely reduced dentitions: a 5-year clinical outcome study. Int J Prosthodont. 2012;25(3):217. 22545250

[5] Verma R, Joda T, Bragger U, Wittneben JG. A systematic review of the clinical performance of tooth-retained and implant-retained double crown prostheses with a follow up of >/= 3 years. J Prosthodont. 2013;22(1):2–12. 22947104 10.1111/j.1532-849X.2012.00905.x

[6] Kern JS, Hanisch O, Hammächer C, Yildirim M, Wolfart S. Telescopic crowns on implants and teeth: evaluation of A clinical study after 8 to 12 years. Int J Oral Maxillofac Implants. 2019;34(4):977–86. 10.11607/jomi.7204 31107933

[7] Brandt S, Winter A, Weigl P, Brandt J, Romanos G, Lauer HC. Conical zirconia telescoping into electroformed gold: A retrospective study of prostheses supported by teeth and/or implants. Clin Implant Dent Relat Res. 2019;21(2):317–23. 30784167 10.1111/cid.12739

[8] Igarashi K, Katagiri H, Abou-Ayash S, Schimmel M, Afrashtehfar KI. Double-Crown prosthesis retention using polyetherketoneketone (PEKK): an in vitro study. J Prosthodont. 2023;32(2):154–61. 35343624 10.1111/jopr.13512

[9] Groesser J, Sachs C, Heiß P, et al. Retention forces of 14-unit zirconia telescopic prostheses with six double crowns made from zirconia–an in vitro study. Clin Oral Investig. 2014;18:1173–11794. 23963618 10.1007/s00784-013-1093-1

[10] MacEntee MI, Walton JN, Glick N. A clinical trial of patient satisfaction and prosthodontic needs with ball and bar attachments for implant-retained complete overdentures: Three-year results. J Prosthet Dent. 2005;93(1):28–37. 15623995 10.1016/j.prosdent.2004.10.013

[11] Tao X, Xu Z, Lin Z, Wu Q. A completely digital workflow for a maxillary tooth-supported complete overdenture and mandibular telescopic denture to manage the treatment of a patient with hypohidrotic ectodermal dysplasia. J Prosthet Dent. 2025;134(1):17–23. 40118684 10.1016/j.prosdent.2025.02.033

[12] Mohamed AMA, Nawar NH. Strain gauge analysis of the stresses induced by different secondary coping materials in tooth supported telescopic overdentures. Eur J Prosthodont Restor Dent. 2022;30(3):214–22. 34982859 10.1922/EJPRD_2361Mohamed09

[13] Joda T, Bornstein MM, Jung RE, Ferrari M, Waltimo T, Zitzmann NU. Recent trends and future direction of dental research in the digital era. Int J Environ Res Public Health. 2020;17(6):1987. 32197311 10.3390/ijerph17061987 PMC7143449

[14] Turkyilmaz I, Hariri N-H. Four-year outcomes of full-arch fixed dental prostheses using CAD/CAM frameworks: A retrospective review of 15 cases. J Clin Experimental Dentistry. 2018;10(10):e1045. 10.4317/jced.55176 PMC6203912 30386512

[15] Alharbi N, Wismeijer D, Osman RB. Additive manufacturing techniques in prosthodontics: where do we currently stand? A critical review. Int J Prosthodont. 2017;30(5). https://pubmed.ncbi.nlm.nih.gov/28750105/10.11607/ijp.5079 28750105

[16] Ahmed N, Abbasi MS, Haider S, Ahmed N, Habib SR, Altamash S, et al. Fit accuracy of removable partial denture frameworks fabricated with CAD/CAM, rapid prototyping, and conventional techniques: A systematic review. Biomed Res Int. 2021;2021(1):3194433. 34532499 10.1155/2021/3194433 PMC8440078

[17] Schubert O, Reitmaier J, Schweiger J, Erdelt K, Güth J-F. Retentive force of E secondary crowns on zirconia primary crowns over time. Clin Oral Invest. 2019;23:2331–8. 10.1007/s00784-018-2657-x 30293185

[18] Kim W, Li XC, Bidra AS. Clinical outcomes of implant-supported monolithic zirconia crowns and fixed partial dentures: a systematic review. J Prosthodont. 2023;32(2):102–7. 35929416 10.1111/jopr.13575

[19] Merk S, Wange C, Stock V, Echberger M, Schmildin P, Roos M, Stawarczyk B. Suitability of secondary PEEK telescopic crown on zirconia primary crown: the influence of fabrication method and Tyaper. J Mater. 2016;9:1–9. 10.3390/ma9110908 PMC5457267 28774027

[20] Schimmel M, Walther M, Al-Haj Husain N, Igarashi K, Wittneben J, Abou-Ayash S. Retention forces between primary and secondary CAD/CAM manufactured telescopic crowns: an in vitro comparison of common material combinations. Clin Oral Investig. 2021;25(11):6297–307. 33834311 10.1007/s00784-021-03928-2 PMC8531068

[21] Stawarczyk B, Ozcan M, Schmutz F, Trottmann A, Roos M, Hammerle CH. Two-body wear of monolithic, veneered and glazed zirconia and their corresponding enamel antagonists. Acta Odontol Scand. 2013;71:102–12. 22364372 10.3109/00016357.2011.654248

[22] Rojas-Vizcaya F. Full zirconia fixed detachable implantretained restorations manufactured from monolithic zirconia: clinical report after two years in service. J Prosthodont. 2011;20:570–6. 22003832 10.1111/j.1532-849X.2011.00784.x

[23] Gentz FI, Brooks DI, Liacouras PC, et al. Retentive forces ofremovable partial denture clasp assemblies made frompolyaryletherketone and cobalt-chromium: a comparative study. J Prosthodont. 2022;31:299–304. 34081360 10.1111/jopr.13398

[24] Alqurashi H, Khurshid Z, Syed AUY, Rashid Habib S, Rokaya D, Zafar MS. Polyetherketoneketone (PEKK): an emerging biomaterial for oral implants and dental prostheses. J Adv Res. 2020;28:87–95. 33384878 10.1016/j.jare.2020.09.004 PMC7770505

[25] Zol SM, Alauddin MS, Said Z, Mohd Ghazali MI, Hao-Ern L, Mohd Farid DA, Zahari NAH, Al-Khadim AHA, Abdul Aziz AH. Description of Poly(aryl-ether-ketone) materials (PAEKs), polyetheretherketone (PEEK) and polyetherketoneketone (PEKK) for application as a dental material. Mater Sci Rev Polym (Basel). 2023;15(9):2170. 10.3390/polym15092170 PMC10180673 37177316

[26] Bandela V, Kanaparthi S. Finite element analysis and its appli cations in dentistry. In: Baccouch M, editor. Finite element methods and their applications. London, UK: IntechOpen; 2020. pp. 1–24.

[27] Al-Kordy NM, Al-Saadi MH. Finite element study of stress distribution with tooth-supported mandibular overdenture retained by ball attachments or resilient telescopic crowns. Eur J Dentistry. 2023;17(02):539–47. 10.1055/s-0042-1749363 PMC10329555 36351452

[28] Amornvit P, Rokaya D, Keawcharoen K, Thongpulsawasdi N. Stress distribution in implant retained finger prosthesis: a finite element study. J Clin Diagn Research: JCDR. 2013;7(12):2851. 24551656 10.7860/JCDR/2013/7001.3775 PMC3919359

[29] Liliana S, Florin T, Sorin P. Finite element study on Corono radicular restored teeth. Inter J Model Optim. 2012;2(03):342–3452.

[30] Asundi A, Kishen A. A strain gauge and photoelastic analysis of in vivo strain and in vitro stress distribution in human dental supporting structures. Arch Oral Biol. 2000;45(7):543–50. 10785517 10.1016/s0003-9969(00)00031-5

[31] Srirekha A, Bashetty K. Infinite to finite: an overview of finite element analysis. Indian J Dent Res. 2010;21(03):425–32. 20930357 10.4103/0970-9290.70813

[32] Wang Y, Chen L. Advancements in finite element analysis for prosthodontics. Prog Med Devices. 2024;2(4):189–204.

[33] Fischer CAI, Ghergic DL, Vranceanu DM, et al. Assessment of force retention between milled metallic and ceramic telescopic crowns with different taper angles used for oral rehabilitation. Mater (Basel). 2020;13(21):4814. 10.3390/ma13214814 . Published 2020 Oct 28. 10.3390/ma13214814 PMC7663722 33126581

[34] Wagner C, Stock V, Merk S, et al. Retention load of telescopic crowns with different taper angles between Cobalt-Chromium and polyetheretherketone made with three different manufacturing processes examined by Pull-Off test. J Prosthodont. 2018;27(2):162–8. 10.1111/jopr.12482. 27037795 10.1111/jopr.12482

[35] Igarashi K, Katagiri H, Abou-Ayash S, Schimmel M, Afrashtehfar KI. Double‐crown prosthesis retention using polyetherketoneketone (PEKK): an in vitro study. J Prosthodont. 2023;32(2):154–61. 35343624 10.1111/jopr.13512

[36] Tribst JP, Dal Piva AM, Syed AU, Alrabiah M, Al-Aali KA, Vohra F, Abduljabbar T. Comparative stress analysis of polyetherketoneketone (PEKK) telescopic crowns supported by different primary crown materials. Appl Sci. 2022;12(7):3446.

[37] Idzior-Haufa M, Pilarska AA, Hędzelek W, Boniecki P, Pilarski K, Dorocka-Bobkowska B. A Comparison of Biomechanical Properties of Implant-Retained Overdenture Based on Precision Attachment Type. Materials (Basel). 2021. Cited 2025. 10.3390/ma14102598 PMC8155947 34067572

[38] Shahmiri R, Das R, Aarts JM, Bennani V. Finite element analysis of an implant-assisted removable partial denture during bilateral loading: occlusal rests position. J Prosthet Dent. 2014;112(5):1126–33. Cited 16 May 2025. 10.1016/j.prosdent.2014.04.023 24951387

[39] Natali AN, Pavan PG, Ruggero AL. Analysis of bone–implant interaction phenomena by using a numerical approach. Clin Oral Implants Res. 2006;17(1):67–74. 10.1111/j.1600-0501.2005.01162.x. 16441786 10.1111/j.1600-0501.2005.01162.x

[40] Tiossi R, Faria ACL, Lin CL, Ribeiro RF, Greghi SLA, Rodrigues RCS. Validation of finite element models for strain analysis of implant-supported prostheses using digital image correlation. Dent Mater. 2013;29(7):788–96. 10.1016/j.dental.2013.04.010. 23694844 10.1016/j.dental.2013.04.010

[41] Baccouch Mahboub. Finite element methods and their applications. 2021. Cited 16 May 2025; Available from: https://books.google.com/books/about/Finite_Element_Methods_and_Their_Applica.html?id=o7ZaEAAAQBAJ.

[42] Stock V, Wagner C, Merk S, Roos M, Schmidlin PR, Eichberger M et al. Retention force of differently fabricated telescopic PEEK crowns with different tapers. Dent Mater J. 2016;35(4):594–600. Cited 12 Jun 2025. Available from: https://europepmc.org/article/med/27477224. 10.4012/dmj.2015-249 27477224

[43] Emera RM, Altonbary GY, Elbashir SA. Comparison between all zirconia, all PEEK, and zirconia-PEEK telescopic attachments for two implants retained mandibular complete overdentures: in vitro stress analysis study. J Dent Implants. 2019;9(1):24–9.

[44] Kamel A, Badr A, Fekry G, Tsoi J. Parameters affecting the retention force of CAD/CAM telescopic crowns: A focused review of in vitro studies. J Clin Med. 2021;10(19):4429–4429. 34640446 10.3390/jcm10194429 PMC8509650

[45] Chen Y, Liu W, Wu Z, Wang S, Li Y, Su B, Li S. Advantages and feasibility of prefabricated PEEK crowns for aesthetic restoration in primary teeth. Sci Rep. 2024;14(1):28398. 39551812 10.1038/s41598-024-79306-1 PMC11570650

[46] Mostafa D, Hussein O, Hussein MO. Biomechanical Performance of PEEK and Graphene-Modified PMMA as Telescopic Removable Partial Denture Materials: A Nonlinear 3D Finite Element Analysis. 2022. 10.11607/ijp.8177 36125879

[47] Papathanasiou I, Kamposiora P, Papavasiliou G, Ferrari M. The use of PEEK in digital prosthodontics: A narrative review. BMC Oral Health. 2020;20(1). 10.1186/s12903-020-01202-7 PMC7398079 32741366

[48] Niem T, Youssef N, Wöstmann B. Energy dissipation capacities of CAD-CAM restorative materials: A comparative evaluation of resilience and toughness. J Prosthet Dent;121(1):101–9. 10.1016/j.prosdent.2018.05.0. 10.1016/j.prosdent.2018.05.003 30017162

[49] Han K, Lee J, Shin SW, Han K, Lee J, Shin SW. Implant-and tooth-supported fixed prostheses using a high-performance polymer (Pekkton) framework. Int J Prosthodont. 2016;29:451–4. 27611747 10.11607/ijp.4688

[50] Aboelnagga MM. Comparative stress analysis of BioHPP and PEKK CAD/CAM frameworks in mandibular All-on-Four fixeddetachable prosthesis on its supporting implants. ASDJ. 2023;32:6–17.

[51] Mizusawa K, Shin C, Okada D, Ogura R, Komada W, Saleh O, Huang L, Miura H. The investigation of the stress distribution in abutment teeth for connected crowns. J Dent Sci. 2021;16(3):929–36. 34141107 10.1016/j.jds.2020.11.005 PMC8189894

[52] Chang HH, Yeh CL, Wang YL, Huang YC, Tsai SJ, Li YT, et al. Differences in the Biomechanical behaviors of natural teeth and dental implants. Dent Mater. 2021;37(4):682–9. 33589270 10.1016/j.dental.2021.01.003

[53] Száva DT, Száva A, Száva J, Gálfi B, Vlase S. Dental implant and natural tooth Micro-Movements during Mastication—In vivo study with 3D VIC method. J Pers Med. 2022;12(10). https://pubmed.ncbi.nlm.nih.gov/36294829/. 10.3390/jpm12101690 PMC9605270 36294829

[54] Thaungwilai K, Tantilertanant Y, Tomeboon P, Singhatanadgit W, Singhatanadgid P. Biomechanical evaluation of stress distribution in a natural tooth adjacent to a dental implant using finite element modeling. Eur J Gen Dent. 2025. 10.1055/s-0044-1800841.

[55] Robinson D, Aguilar L, Gatti A, Abduo J, Lee PVS, Ackland D. Load response of the natural tooth and dental implant: A comparative biomechanics study. J Adv Prosthodont. 2019;11(3):169–78. 31297176 10.4047/jap.2019.11.3.169 PMC6609758

[56] Ulbrich NL, Hecke MB, Possobom AL, Bassanta AD. Comparative FEM analysis of the stresses transmitted by intramobile elements of the IMZ implant. Comput Methods Biomech Biomedical Eng – 2. 2020;721–8. https://www.taylorfrancis.com/chapters/edit/10.1201/9781003078289-95/comparative-fem-analysis-stresses-transmitted-intramobile-elements-imz-implant-ulbrich-hecke-possobom-bassanta

[57] Ibrahim CR, Sameh A, Askar O. A finite element analysis study on different angle correction designs for inclined implants in All-On-Four protocol. BMC Oral Health. 2024;24:331. 10.1186/s12903-024-04091-2. 38481220 10.1186/s12903-024-04091-2 PMC10938696

[58] Lofaj F, Kučera J, Németh D, Kvetková L. Finite element analysis of stress distributions in mono- and bi-cortical dental implants. Mater Sci Eng C Mater Biol Appl. 2015;50:85–96. Epub 2015 Feb 2. PMID: 25746249. 25746249 10.1016/j.msec.2015.01.095

[59] Ceddia M, Romasco T, De Bortoli N Jr., Mello BF, Piattelli A, Mijiritsky E, Di Pietro N, Trentadue B. Biomechanical finite element analysis of two types of Short-Angled implants across various bone classifications. Materials. 2024;17:5680. 10.3390/ma17235680. 39685119 10.3390/ma17235680 PMC11642249

[60] Pedroso JM, Enger M, Bandeira P, Magalhães FD. Comparative study of friction and wear performance of PEK, PEEK and PEKK binders in tribological coatings. Polymers. 2022;14:4008. https://www.mdpi.com/2073-4360/14/19/4008 10.3390/polym14194008 PMC9571662 36235956

[61] Ruggiero A, D’Amato R, Sbordone L, Haro FB, Lanza A. Experimental comparison on dental biotribological pairs Zirconia/Zirconia and Zirconia/Natural tooth by using a reciprocating tribometer. J Med Syst. 2019;43(4):97. 30868440 10.1007/s10916-019-1230-8

[62] Ciocan LT, Ghitman J, Vasilescu VG, Iovu H. Mechanical properties of Polymer-Based blanks for machined dental restorations. Materials. 2021;14(23):7293. 34885448 10.3390/ma14237293 PMC8658077

[63] Chen X, Mao B, Zhu Z, Yu J, Lu Y, Zhang Q, et al. A three-dimensional finite element analysis of mechanical function for 4 removable partial denture designs with 3 framework materials: CoCr, Ti-6Al-4V alloy and PEEK. Sci Rep. 2019;27(1):1–10. 10.1038/s41598-019-50363-1 PMC6765051 31562391

[64] Elsayed A, Farrag G, Chaar M, Abdelnabi N, Kern M. Influence of different CAD/CAM crown materials on the fracture of Custom-Made titanium and zirconia implant abutments after artificial aging. Int J Prosthodont. 2018;32(1):91–6. 10.11607/ijp.6137 30677119

